# A short walk in quantum probability

**DOI:** 10.1098/rsta.2017.0226

**Published:** 2018-03-19

**Authors:** Robin Hudson

**Affiliations:** Department of Mathematical Sciences, Loughborough University, Loughborough, LE11 3TU, UK

**Keywords:** quantum probability, quantum central limit theorem, quantum planar Brownian motion

## Abstract

This is a personal survey of aspects of quantum probability related to the Heisenberg commutation relation for canonical pairs. Using the failure, in general, of non-negativity of the Wigner distribution for canonical pairs to motivate a more satisfactory quantum notion of joint distribution, we visit a central limit theorem for such pairs and a resulting family of quantum planar Brownian motions which deform the classical planar Brownian motion, together with a corresponding family of quantum stochastic areas.

This article is part of the themed issue ‘Hilbert’s sixth problem’.

## 1. Introduction

Kolmogorov’s great book [1] that provided a rigorous mathematical foundation for the classical theory of probability was published in 1933.

In Kolmogorovian probability theory, as refined by his disciples, the fundamental notion is that of a probability space 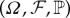, that is, a triple comprising a non-empty set *Ω*, a *σ*-field 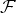 of subsets of *Ω*, and a probability measure 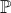 on 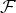. Real-valued random variables, or *observables* in physicists’ language, are represented as real-valued functions *F* on *Ω* which are measurable with respect to the pair of *σ*-fields 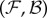, where 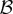 is the *σ*-field of Borel subsets of 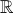, so that 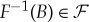 for each 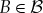.

Such random variables admit a functional calculus; for a bounded, 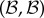-measurable 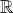-valued function *f* on 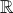, *f*(*F*) is the random variable given by ( *f*(*F*))(*ω*)=*f*(*F*(*ω*)). This extends to complex-valued functions on 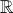 by separating real and imaginary parts. The usual rules of functional calculus hold, e.g.

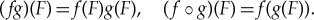


The *probability distribution* of *F* can be defined as the probability measure on the Borel subsets of 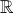 given by

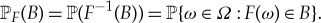
Thus, if it exists, the expectation or mean 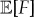 of *F* is given by

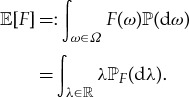
The probability distribution is characterized by the family of expectations it yields for bounded measurable functions *f* of *F*:

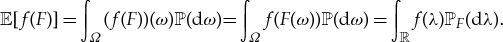
In fact, the exponential functions are sufficient for this characterization, hence the *characteristic functionϕ*_*F*_ of *F*

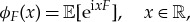
is well named.

One can imagine that, if Kolmogorov had been familiar with another book [2] published slightly earlier, he might have wished to have written a different book. But we must assume that he was unaware of the existence of *quantum probability*.

Quantum probability is, in the first place, a non-commutative extension of classical probability, in which random variables are represented as self-adjoint operators *S* acting in a complex Hilbert space 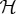, and the underlying probability measure by a unit vector 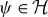. The pair 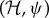 is an example of a *quantum probability space* (but a more general notion will be needed below). According to the spectral theorem, *S* admits a unique spectral resolution,

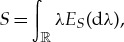
in terms of a projection-valued measure *E*_*S*_ on the Borel field. Using this, the quantum random variable *S* acquires a probability distribution
1.1
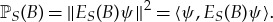
The expectation, if it exists, is given by

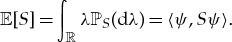
Alternatively, using the bounded Borel measurable functional calculus for *S*,

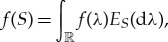
we find that

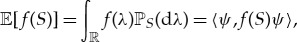
which is always well defined. Hence, we can write the corresponding characteristic function as

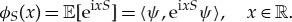


Kolmogorovian classical probability based on a probability space 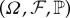 can be included in the quantum framework by taking the complex Hilbert space 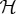 to be 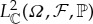 and the unit vector *ψ* to be the element *ψ*(*ω*)=1, *ω*∈*Ω*. The classical random variable *F* can then be represented by the self-adjoint operator mult_*F*_ of multiplication by *F* acting on the domain on which this action yields a function still within 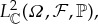 which is the whole of 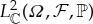 if *F* is bounded.

Conversely, any individual quantum random variable *S* can be realized classically by the function *F*(λ)=λ on the probability space 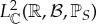, where 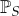 is the probability distribution (1.1).

Now suppose we are given two quantum random variables *R* and *S*. If these are bounded self-adjoint operators, then by definition they commute if *RS*=*SR*. This is the case if and only if any of the following three equivalent conditions hold.

— for arbitrary Borel sets *A* and *B*

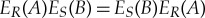
— for arbitrary bounded Borel measurable functions *f* and *g*

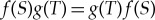
— for arbitrary real *x* and *y*

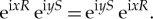

For unbounded *R* and *S*, we define commutativity to mean that these three equivalent conditions hold. We may then define the joint probability distribution of the commuting random variables *R* and *S* as the probability measure 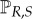 on 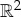 for which

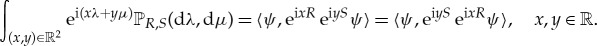
That such 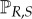 exists and is unique can be inferred from the two-dimensional form of Bochner’s theorem, after checking that the function *ϕ*_*R*,*S*_(*x*,*y*)=〈*ψ*,e^*ixR*^ e^i*yS*^*ψ*〉 satisfies the conditions

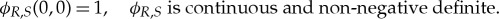


Generalizing the case of a single random variable, the two commuting quantum random variables *R* and *S* can be realized classically by the functions *D*(λ,*μ*)=λ and *F*(λ,*μ*)=*μ* on the probability space 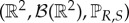.

But if *R* and *S* do not commute, there is usually no sensible notion of joint probability distribution and they cannot be simultaneously realized classically. In quantum mechanics, it is not possible to measure simultaneously the values of the observables represented by *R* and *S*. Thus, there is no way of empirically constructing a joint probability distribution and no obligation on quantum probability to say what it will be. Quantum probability really is different.

In quantum probability, the observables belonging to a particular physical system are often taken to be the self-adjoint operators affiliated to a von Neumann algebra 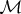, that is, to a unital sub-*algebra of the algebra 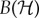 of bounded operators on the Hilbert space 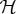, which is closed in the strong operator topology in which a sequence 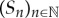 converges to a limit *S* if and only if 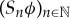 converges to *Sϕ* for every 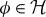. Here, a self-adjoint operator *S* is *affiliated* to 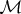 if and only if either it is bounded and belongs to 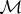 or (equivalently in the bounded case) all its spectral projections belong to 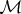 (or 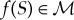 for every bounded measurable function *f*, or 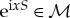 for all 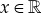). Another assumption which is commonly made, though it does not hold in some important examples, is that the unit vector *ψ* is *cyclic*, that is, 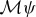 is dense in 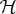, and *separating*, that is, *Sψ*=0 for 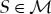 implies that *S*=0.

A *commutative* von Neumann algebra is always isomorphic to the algebra of bounded measurable complex-valued functions acting by multiplication on the Hilbert space 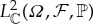 for some probability space 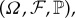 thus quantum probability is in a very precise sense a non-commutative generalization of Kolmogorovian probability. This situation may be compared with non-commutative geometry [3].

## 2. Canonical pairs

Much of my life has been concerned with probabilistic aspects of the Heisenberg commutation relation

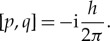
Here, *q* is the position observable and *p* is the canonically conjugate momentum observable of a particle localizable in one dimension, and *h* is Planck’s constant, whose value in mks units is *h*=6.6261103×10^−34^. Despite the smallness of this number, in order to harmonize some probabilistic conventions with physics, we will find it convenient to take *h*=4*π* and to define a *canonical pair* as a pair of self-adjoint operators (*p*,*q*) satisfying the commutation relation
2.1
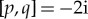
in the sense that the corresponding families of unitary operators 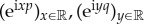 satisfy the formally equivalent but mathematically rigorous Weyl commutation relations
2.2
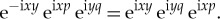


An example of such a pair, called the *Schrödinger pair* (*p*_Schr_,*q*_Schr_), can be constructed in the Hilbert space 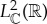 by defining the two families of operators

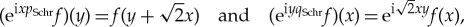
having first verified that the families of unitary operators defined by these actions are indeed both continuous unitary representations of the group 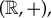 so that by Stone’s theorem they determine unique self-adjoint operators *p*_Schr_ and *q*_*Schr*_, and that (2.2) holds.

In fact, by the Stone–von Neumann uniqueness theorem [4–6], the Schrödinger pair is essentially unique. More precisely given an arbitrary canonical pair (*p*,*q*) acting in a Hilbert space 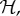 there exists a Hilbert space 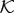 and a Hilbert space isomorphism (i.e. a unitary transformation) *U* from 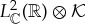 to 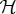, which intertwines each e^i*xp*^ with 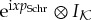 and each e^i*xq*^ with 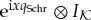.

Another useful canonical pair is in the Hilbert space 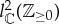 of square-summable complex sequences, which it is convenient to regard as the Fock space over the Hilbert space 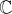, in the sense of the following definition.

**Definition 2.1.**

Given a complex Hilbert space 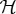, the *Fock space* 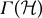 over 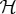 is a Hilbert space equipped with generating a family of *exponential vectors* 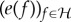 satisfying

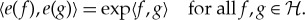

That such a Hilbert space exists and is unique in the sense that, given any two candidate Fock spaces over 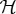, there is a unique Hilbert space isomorphism which exchanges the two candidate exponential vectors corresponding to each 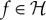 follows from the kernel theorem [7] and the non-negative definiteness of the kernel 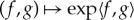 over 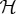.

Physicists usually realize the Fock space explicitly as the infinite direct sum of symmetrized tensor products,
2.3
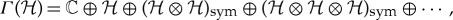
in which case the exponential vectors are given by

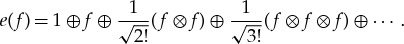
But our more abstract view of Fock spaces lends itself better to probabilistic aspects.

By the uniqueness property, given a Hilbert space automorphism *U* on 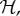 there exists a unique automorphism *Γ*(*U*) on 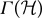, called, for physical reasons, the *second quantization* of *U*, such that, for each exponential vector *e*( *f*), *Γ*(*U*)*e*( *f*)=*e*(*Uf*). Another useful family of unitary operators on 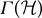 are the so-called *Weyl operators* 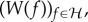 which are conveniently defined by their actions on the exponential vectors,

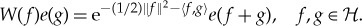
They satisfy the Weyl relation
2.4
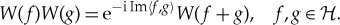


Now take 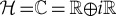 and consider the one-parameter groups of unitary operators 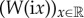 and 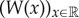. From (2.4)

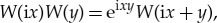
whereas

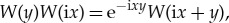
so that the Weyl relations (2.2) hold,

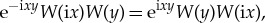
and we obtain a new canonical pair (*p*_Fock_,*q*_Fock_) by writing

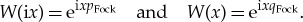


In fact, the pair (*p*_Fock_,*q*_Fock_) is irreducible and the isomorphism *U* of the Stone–von Neumann uniqueness theorem is thus from 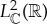 itself to 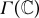. It can be constructed explicitly as follows. First, apply the isomorphism from 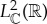 to the Hilbert space 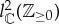 determined by the orthonormal basis of Hermite functions. Then compose this with the isomorphism from 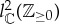 to 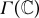 in which the successive tensor powers of 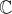 are all identified with 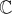 itself by multiplication of complex numbers, and the preimage of each exponential vector is its physicists’ realization,

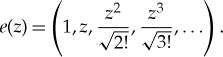


## 3. Joint distributions for canonical pairs

Can we construct a joint probability distribution for a canonical pair (*p*,*q*) acting in a quantum probability space 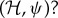 One tempting route to such a construction is based on the observation that, for all real *x* and *y*, the self-adjoint operator *xp*+*yq* can be defined unambiguously as the generator of the one-parameter group 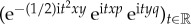. Hence, the unitary operator e^i(*xp*+*yq*)^ is well defined. We can then write 〈*ψ*,e^*i*(*xp*+*yq*)^*ψ*〉 as a Fourier–Stieltjes transform,

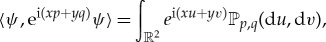
and hope that 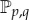 is a plausible joint distribution.

Sometimes, this works [8]; sometimes, it does not [9]. To see this, let us first write

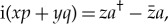
where *z*=*x*+*iy* and *a*^†^ and *a* are the so-called *creation* and *annihilation* operators, respectively,

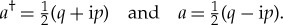
By formal manipulation using the Baker–Hausdorff formula (which can be made rigorous [7]), one finds that

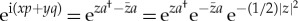
and so
3.1
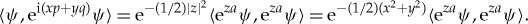


Now, using the Schrödinger realization, (*p*,*q*)=(*p*_Schr_,*q*_Schr_), take *ψ* to be the zero-order Hermite function in 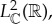 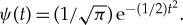 Then,

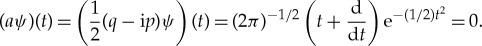
So, e^*za*^*ψ*=*ψ* and it follows from (3.1) that our candidate characteristic function reduces to

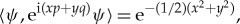
corresponding to a joint distribution of isotropic Gaussian form with unit variance.

But, if *ψ*_1_=*a*^†^*ψ* we find that

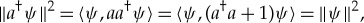
so *ψ*_1_ is a unit vector which we can use to generate probabilities, and, using the commutation relation e^*za*^*a*^†^=(*a*^†^+*z*)e^*za*^ and the relation 〈*a*^†^*ψ*,*ψ*〉=〈*ψ*,*aψ*〉=0,

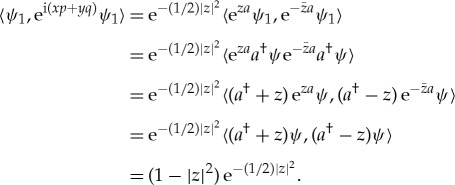


This is the Fourier transform of

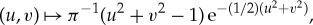
which may perhaps look like a plausible joint probability density until one uses it to compute the probability that (*p*,*q*) lies in the disc {(*u*,*v*):*u*^2^+*v*^2^≤1}.

In fact, the appearence of such negative probabilities is the rule rather than the exception; in the Schrödinger realization, only when the probability vector is itself of essentially Gaussian form

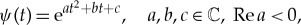
do they not appear [9]. Nowadays, the ‘negative probabilities’ which always appear otherwise are widely used in quantum optics as a measure of ‘quantumness’.

To find a truly quantum substitute for the joint probability distribution of a canonical pair (*p*,*q*), recall that, in the commutative case, the joint probability distribution encodes all expectations of bounded measurable functions of the two observables, that is, elements of the von Neumann algebra that they generate. Let us find a way of similarly encoding the expectations of elements of the von Neumann algebra 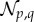 generated by a canonical pair (*p*,*q*). In accordance with the Stone–von Neumann theorem, we write

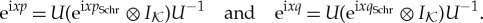


Then, because the Schrödinger representation is irreducible,

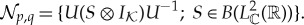
For each element, we write

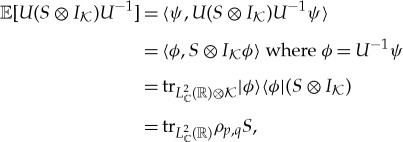
where *ρ*_*p*,*q*_ is the partial trace 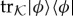 of the one-dimensional projector |*ϕ*〉〈*ϕ*| over the auxiliary Hilbert space 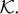 The operator *ρ*_*p*,*q*_ is a non-negative trace-class operator of unit trace on 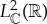 called the *distribution operator* of (*p*,*q*). It is our substitute for a classical joint probability distribution for *p* and *q*. Thus, two canonical pairs are *identically distributed* if they have the same distribution operator.

Now suppose that, given two canonical pairs (*p*,*q*) and (*p*^′^,*q*^′^) which commute with each other in the sense that, for arbitrary real *x*,*y*, each of *p* and *q* commute with each of *p*^′^ and *q*^′^,
3.2
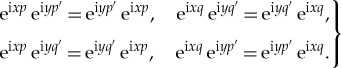
Equivalently, the two pairs generate von Neumann algebras contained in each other’s commutants. The two-dimensional form of the Stone–von Neumann theorem allows us to define, in a similar way, a joint distribution operator *ρ*_*p*,*q*,*p*^′^,*q*^′^_, which is an operator on 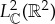 which encodes the expectations of elements of the von Neumann algebra generated jointly by *p*,*q*,*p*^′^,*q*^′^. The two pairs are *stochastically independent* if *ρ*_*p*,*q*,*p*^′^,*q*^′^_=*ρ*_*p*,*q*_⊗*ρ*_*p*^′^,*q*^′^_, in so far as 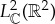 is canonically identified with 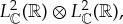 or equivalently if and only if 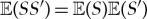 for arbitrary *S* and *S*^′^ belonging to the von Neumann algebras generated by the pairs (*p*,*q*) and (*p*^′^,*q*^′^), respectively.

## 4. A quantum central limit theorem

Let 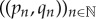 be a sequence of canonical pairs, any two of which commute with each other in the sense of (3.2). Then it makes sense to demand also that they are independent and identically distributed. Assuming also that the means are zero, 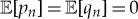, and that the second moments are all finite,

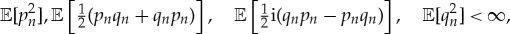
then by applying a common unimodular linear transformation

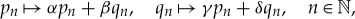
where 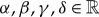 with *αδ*−*βγ*=1, we may assume without loss of generality that the covariance matrix takes the canonical form
4.1
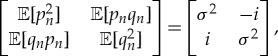
where we define

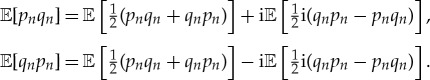
The variance parameter *σ*^2^≥1 in view of the Heisenberg uncertainty inequality.

The sequence 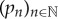 consists of mutually commuting independent, identically distributed random variables of mean 0 and variance *σ*^2^. So by the classical Demoivre–Laplace central limit theorem, the sequence 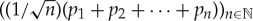 converges in distribution to the normal limit distribution *N*(0,*σ*^2^) of mean zero and variance *σ*^2^. Similarly, 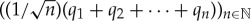 converges in distribution to *N*(0,*σ*^2^).

So far, so classical. Now consider the commutator

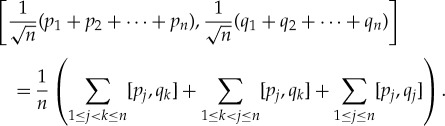
By our mutual commutativity assumption, the first two sums vanish, leaving only

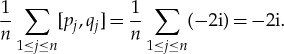
So, for each *n*=1,2,…,
4.2
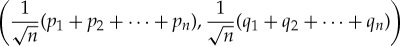
is another canonical pair! The reader is invited to rigorously reformulate and prove this statement, using only the one-parameter groups generated by the various unbounded self-adjoint operators.

Is there a *quantum* central limit theorem behind this? Note that such a theorem is not just the two-dimensional version of the de Moivre–Laplace theorem as in general the canonical pairs (4.2) do not have a joint distribution in the classical sense.^1^

Let us first construct the limit distribution operator, which must have the properties that the individual distributions of *p* and *q* are both *N*(0,*σ*^2^) distributed, and that the covariance matrix is given by (4.1), which is inherited unchanged from each of the approximands (4.2). First, we consider the case of minimal variance allowed by the Heisenberg uncertainty principle, namely *σ*^2^=1. In this case, the distribution operator is the ‘pure state density operator’ *ρ*_*p*,*q*_=|*ψ*_0_〉〈*ψ*_0_|, where 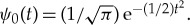 Indeed, passing to the equivalent Fock representation, we find that, for 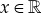,

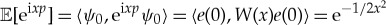
so that *p* (and similarly *q*) is indeed *N*(0,1) distributed. Moreover,

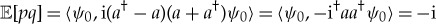
and similarly 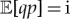.

Now consider the case *σ*^2^>1. Define positive real numbers *α* and *β* by
4.3
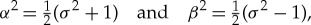
so that *σ*^2^=*α*^2^+*β*^2^ and *α*^2^−*β*^2^=1. Given a complex Hilbert space *h*, denote by 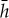 the Banach dual space of *h*, for *f*∈*h* by 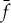 the bounded linear functional 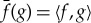 and, for *S*∈*B*(*h*), by 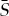 the operator 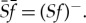 Now, equip the Hilbert space tensor product 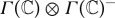 with the unit vector 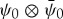 and define a canonical pair (*p*_*σ*_,*q*_*σ*_) informally by

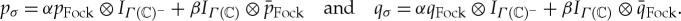
The random variable *p*_*σ*_ is the sum of *independent*mutually commuting random variables 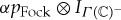 and 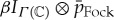, which are both normally distributed with zero mean and with variances *α*^2^ and *β*^2^, respectively. Hence, *p*_*σ*_ is *N*(0,*α*^2^+*β*^2^), that is, *N*(0,*σ*^2^). The same is true of *q*_*σ*_. Moreover,

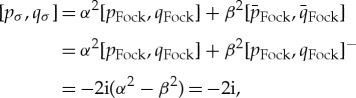
so (*p*_*σ*_,*q*_*σ*_) is indeed a canonical pair as claimed. Finally, because the means are zero

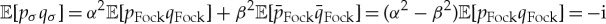
and similarly 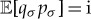.

What does it mean to say that the sequence of canonical pairs (4.2) converges in distribution to (*p*_*σ*_,*q*_*σ*_)? The simplest notion of convergence is that of distribution operators in the Hilbert–Schmidt norm. This would correspond to convergence in the *L*^2^ sense of the densities of joint distributions to the corresponding Gaussian density, in contrast with the ‘weak convergence’ in the probabilistic sense [10] of the two-dimensional classical central limit theorem. But it can be shown that [11] in the quantum case convergence is in the stronger sense, for an arbitrary bounded operator *S* on 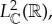


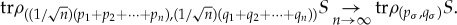
As bounded operators are non-commutative ‘bounded measurable functions’, our quantum central limit theorem, in which convergence is in the sense of expectations of such ‘functions’, is actually stronger than the classical central limit theorem in which only expectations of bounded *continuous* functions must converge. This is just as well because it is not obvious how to find an explicit non-commutative substitute in this context for the continuous functions similar to the von Neumann algebra generated for the measurable ones.

For many years, the quantum central limit theorem of [11] found few applications, though as we will see below it was crucial in suggesting theoretical advances such as quantum Brownian motion. Recently, however, it has found an increasing number of applications in quantum statistics and estimation problems and elsewhere [12,13].

## 5. Quantum planar Brownian motion [14]

Donsker’s theorem [10], also known variously as the functional central limit theorem and as the^2^ invariance principle, is a generalization of the well-known construction of Brownian as a limit of Bernoulli random walks.

**Theorem 5.1.**

*Let* 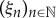 *be a sequence of independent identically distributed random variables of mean zero and variance σ*^2^*. Then the sequence* 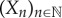 *of random processes on* [0,1]

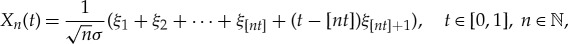
*converges weakly to a standard one-dimensional Brownian motion X.*

Here, weak convergence is in the sense of probability measures on the metric space of continuous functions vanishing at 0 on [0,1] equipped with the sup norm. In particular, bounded continuous functions of *X*_*n*_ such as the supremum converge with *n* to their values on *X*. Thus, the convergence is stronger than that of all multi-dimensional joint distributions; for example, the supremum depends on infinitely many values.

Now let 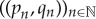 be a sequence of independent identically distributed canonical pairs. By the invariance principle, we can expect informally that, individually, each of the sequences of processes 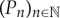 and 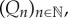 where

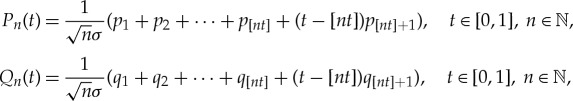
converge to Brownian motions *P* and *Q*, respectively. To what do they converge jointly?

To answer this question, let us observe that, as follows from (2.1),

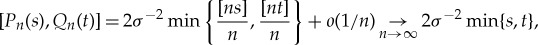
suggesting that the limit processes *P* and *Q* should satisfy
5.1
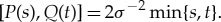
How do we construct two unit variance Brownian motions satisfying this commutation relation?

Consider first the case of minimal variance, *σ*^2^=1. We work in the Fock space 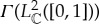.^3^ Fix a real parameter *θ*∈[0,2*π*[. Let *χ*_[0,*t*[_ denote the indicator function of [0.*t*[⊂[0,1] and consider the family of Weyl operators 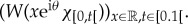 As

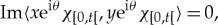
the Weyl relation (2.4) gives the one-parameter group property

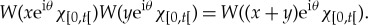
So, for each fixed *θ*∈[0,2*π*[ and *t*∈[0,1], there is a unique self-adjoint operator *Ξ*_*θ*_(*t*)

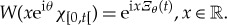
As similarly Im〈*x*e^i*θ*^*χ*_[0,*s*[_,*y*e^i*θ*^*χ*_[0,*t*[_〉=0, the family (*Ξ*_*θ*_(*s*))_*s*∈[0,1]_ is commutative. Using the explicit action of the Weyl operators *e*(0), it can be verified that, with this probability vector, the process *Ξ*_*θ*_ is Gaussian, with stationary independent increments, and that 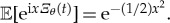 This suffices for us to recognize it as a standard (unit variance) Brownian motion.

Now define processes *P* and *Q* by *P*=*Ξ*_*π*/2_,*Q*=*Ξ*_0_. Then,

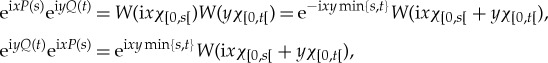
so

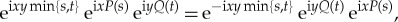
which, as required, is the rigorous Weyl form of (5.1) with *σ*^2^=1. Note that we could equally well have taken *P*=*Ξ*_*π*/2+*θ*_, *Q*=*Ξ*_*θ*_. Denoting the latter candidates by *P*_*θ*_ and *Q*_*θ*_, it can be shown that this unit variance *quantum planar Brownian motion* has rotational invariance

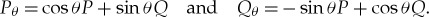


When *σ*^2^>1, we construct (*P*,*Q*) in the Hilbert space 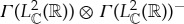 by defining the corresponding one-parameter unitary groups as

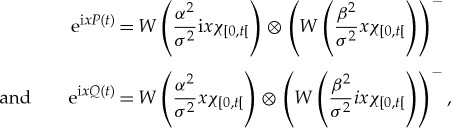
where *α* and *β* are defined by (4.3), and taking the unit probability vector as *e*(0)⊗(*e*(0))^−^. Then *P* and *Q* are both sums of two independent Brownian motions of variances *α*^2^/*σ*^2^ and *β*^2^/*σ*^2^, and hence are both Brownian motions of variance *α*^2^/*σ*^2^+*β*^2^/*σ*^2^=1. But, they satisfy (5.1) as required. This may be seen using the Weyl relation and the rules 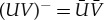 and 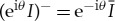.

The resulting quantum planar Brownian motion inherits rotational invariance from the case *σ*^2^=1.

An important joint property of the Brownian motions *P* and *Q* is that, despite their non-commutativity, increments of *P* commute with increments of *Q* over disjoint intervals. Indeed, if 0≤*v*≤*u*≤*t*≤*s*≤1, then, informally,

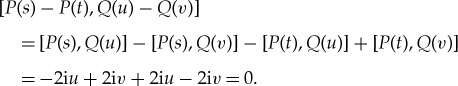
In fact, not only do the increments generate commuting von Neumann algebras but also they are independent in the sense that, for arbitrary real *x*,*y*,

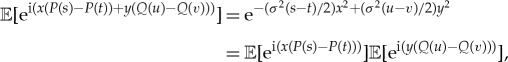
as is seen by ‘splitting’ [7,15] the Fock space 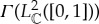 between *u* and *t*. This strong independent increments property leads on to a corresponding genuinely non-commutative strong Markov property for planar quantum Brownian motion [16] and a corresponding splitting at each Markov time [17].

## 6. Quantum Lévy area

The minimal variance quantum planar Brownian motion has found many applications, most of which depend on the corresponding quantum stochastic calculus [7,15]. The case *σ*^2^>1 has so far proved less versatile even though the corresponding quantum stochastic calculus [18,19] is in some ways technically simpler.

One of the most interesting constructions based on classical planar Brownian motion is Lévy’s stochastic area [20]. Informally, this is the signed area between the chord joining two points on the Brownian path and the path itself. Rigorously, it can be constructed either as a martingale limit of polygonal approximations [20], or an equivalent iterated stochastic integral [21]. Though the result can no longer be understood as an area (there is no path), both methods of construction can be imitated for quantum planar Brownian motion [22,23].

The classical Lévy area is related to other areas of mathematics, for example its moments are essentially the well-known Euler numbers which evaluate the Riemann zeta function at even natural numbers. The corresponding quantum moments have been calculated recently [24]; except when *σ*=1 when they all vanish [22], they involve the combinatorial Eulerian numbers, and correctly approach the corresponding classical values in the classical limit as 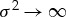.

More generally, the so-called *Lévy area formula* is for the conditional characteristic function
6.1
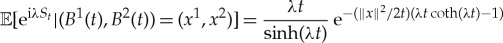
of the Lévy area *S*_*t*_ over the time interval [0,*t*], given the value (*x*^1^,*x*^2^) of the planar Brownian motion (*B*^1^,*B*^2^) at time *t*. It has many such applications [25] and has recently been applied to simplified proofs of Apéry’s celebrated theorem that *ζ*(3) is irrational.

A direct quantum analogue of (6.1) cannot be defined as it involves a joint value of the two mutually non-commuting components of quantum planar Brownian motion. But, using the rotational invariance of the Brownian motion, we can rewrite (6.1) as

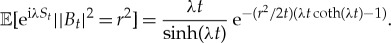
In this form, it is evident that the information in (6.1) is implicitly contained in the joint probability distribution for *S*_*t*_ and the squared Brownian radius |*B*_*t*_|^2^. A quantum Lévy area formula would similarly contain the same information as a joint distribution for the quantum Lévy area 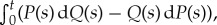 and the quantum-squared Brownian radius *P*(*t*)^2^+*Q*(*t*)^2^. Unfortunately, these two processes do not commute with each other or with themselves at different times.

However, help is at hand in the observation that *P*(*t*)^2^+*Q*(*t*)^2^ is, like the Lévy area, itself an iterated stochastic integral; in fact,

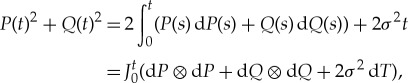
where, following the notation of [22,24], 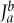 means integrate the multi-differential which follows as an iterated integral over [*a*,*b*]. Having removed the time integral which commutes with the rest, we may find a substitute for the joint characteristic function, which is the exponential of a double integral and thus problematic in a non-commutative context, as the corresponding *double product integral,* to define which does not require commutativity.

A start has been made on this procedure [26].

## Footnotes

1 In fact their Wigner densities are everywhere non-negative for *evenn*, as may be seen using multiplicative properties of the corresponding quasi-characteristic functions.

2 The Demoivre–Laplace central limit theorem is surely the first and therefore most properly ‘the’ invariance principle.

3 The construction which follows is sometimes confused with the so-called *Wiener chaos expansion*, in which every real-valued square-integrable function of a one-dimensional classical Brownian motion *X* has an expansion as a sum of iterated stochastic integrals with real-valued integrands against the Itô integrator *dX*, resulting in a Hilbert-space decomposition analogous to (2.3) but involving only *real* Hilbert spaces.

## Data accessibility

This article has no additional data.
